# Factors associated with effective implementation of screening, brief intervention, and referral to treatment in the emergency department

**DOI:** 10.1186/1940-0640-7-S1-A78

**Published:** 2012-10-09

**Authors:** Alyssa Forcehimes, Cameron Crandall, Michael Bogenschutz, Dennis Donovan, Robert Lindblad, Robrina Walker

**Affiliations:** 1 Center on Alcoholism, Substance Abuse, and Addictions, University of New Mexico, Albuquerque, NM, USA; 2 Department of Emergency Medicine, University of New Mexico, Albuquerque, NM, USA; 3 Department of Psychiatry, University of New Mexico CASAA, Albuquerque, NM, USA; 4 Alcohol & Drug Abuse Institute, University of Washington, Seattle, WA, USA; 5 The EMMES Corporation, Rockville, MD, USA; 6 Department of Psychiatry, University of Texas Southwestern Medical Center, Dallas, TX, USA

## 

Evidence-based screening, brief intervention, and referral to treatment (SBIRT) programs focusing on drug and alcohol use have successfully been implemented in a variety of general medical settings. Such programs require adaptations to function effectively in a high-volume hospital emergency department (ED) setting. We describe implementation and training procedures used in the six-site National Institute on Drug Abuse-Clinical Trials Network (NIDA-CTN) Screening, Motivational Assessment, Referral and Treatment (SMART-ED) trial and present lessons learned from the implementation of the study. The discussion is organized around issues of site selection, staff selection, research assistant and interventionist training, site preparation, and data collection. Several implementation components were particularly important in the SMART-ED trial: 1) Site selection—department and ED staff buy-in was central to decisions on which sites were chosen to participate. 2) Staff selection—interventionists/RAs needed to possess both the empathy necessary to deliver a motivational-interviewing intervention and the research knowledge necessary for protocol adherence. 3) Research assistant and interventionist training and ongoing coaching—in-person and webinar trainings ensured that research staff understood and were able to follow protocol procedures and were certified to deliver the intervention and study procedures that required it. Ongoing coaching based on reviews of the intervention recordings and feedback on compliance with study procedures is successfully preventing drift. 4) Site preparation—prior to beginning the main trial, each site had real-world practice conducting study procedures through standardized patient visits. 5) Data collection—screening data is collected using direct entry into tablet computers to facilitate rapid screening and mobility in the ED setting. There are unique factors associated with effective implementation of SBIRT delivered in the ED. Some of the procedures used in this clinical trial may be useful in the successful implementation of clinical SBIRT programs in EDs.

